# The Polymorphic Membrane Protein G Has a Neutral Effect and the Plasmid Glycoprotein 3 an Antagonistic Effect on the Ability of the Major Outer Membrane Protein to Elicit Protective Immune Responses against a *Chlamydia muridarum* Respiratory Challenge

**DOI:** 10.3390/vaccines11030504

**Published:** 2023-02-21

**Authors:** Anatoli Slepenkin, Sukumar Pal, Steven Hoang-Phou, Abisola Abisoye-Ogunniyan, Amy Rasley, Patrik D’haeseleer, Matthew A. Coleman, Luis M. de la Maza

**Affiliations:** 1 Department of Pathology, Laboratory Medicine, Medical Sciences I, Room D440, University of California, Irvine, CA 92697, USA; 2 Physical and Life Sciences Directorate, Lawrence Livermore National Laboratory, Livermore, CA 94550, USA; 3 Department of Radiation Oncology, School of Medicine, University of California, Davis, CA 95616, USA

**Keywords:** *Chlamydia muridarum*, vaccine, mouse, major outer membrane protein, polymorphic membrane protein G, plasmid glycoprotein 3, adjuvants

## Abstract

*Chlamydia trachomatis* is the most common bacterial sexually transmitted pathogen. The number of chlamydial infections continuous to increase and there is an urgent need for a safe and efficacious vaccine. To assess the ability of the *Chlamydia muridarum* polymorphic membrane protein G (PmpG) and the plasmid glycoprotein 3 (Pgp3) as single antigens, and in combination with the major outer-membrane protein (MOMP) to induce protection, BALB/c mice were immunized utilizing CpG-1826 and Montanide ISA 720 VG as adjuvants. Following vaccination with MOMP, significant humoral and cell-mediated immune responses were observed, while immunization with PmpG, or Pgp3, elicited weaker immune responses. Weaker immune responses were induced with MOMP+Pgp3 compared with MOMP alone. Following the intranasal challenge with *C. muridarum,* mice vaccinated with MOMP showed robust protection against body-weight loss, inflammatory responses in the lungs and number of *Chlamydia* recovered from the lungs. PmpG and Pgp3 elicited weaker protective responses. Mice immunized with MOMP+PmpG, were no better protected than animals vaccinated with MOMP only, while Pgp3 antagonized the protection elicited by MOMP. In conclusion, PmpG and Pgp3 elicited limited protective immune responses in mice against a respiratory challenge with *C. muridarum* and failed to enhance the protection induced by MOMP alone. The virulence of Pgp3 may result from its antagonistic effect on the immune protection induced by MOMP.

## 1. Introduction

*C. trachomatis* is the most common sexually transmitted bacterial pathogen in the world and can also cause ocular, respiratory and gastrointestinal infections [1,2]. *C. trachomatis* infections during pregnancy can significantly affect neonatal outcomes [3]. Although screening programs may have decreased the number of patients who develop long-term sequelae, they have failed to control the spread of this pathogen and the accumulated costs of caring for infected patients continues to increase [4,5,6].

Vaccination trials in humans and non-human primates to protect against *C. trachomatis* (trachoma), using inactivated whole organisms yielded important findings [7,8,9]. Several vaccine formulations were protective for 2–3 years after immunization, and the protection was serovar/serogroup specific. However, upon re-exposure to *Chlamydia*, some individuals developed a hypersensitivity reaction and the possibility that an antigenic component of *Chlamydia* present in these inactivated vaccines mediated these adverse reactions motivated the search for a subunit vaccine [7,9,10,11,12,13,14,15,16,17].

A subunit vaccine that will provide protection against all *C. trachomatis* serovars will require the selection of well-conserved antigens among clinical isolates. The chlamydial major outer membrane protein (MOMP) was initially identified as a potential protective antigen when the serovar/serogroup protection observed during the trachoma vaccine trials was found to correlate with the DNA sequence of this protein [18,19,20]. MOMP is a 40 kDa protein that forms a 120 kDa homotrimer and constitutes ~60% of the outer membrane protein mass of chlamydia. MOMP has four surface-exposed variable domains (VD), and five constant domains (CD) located in β-barrels that cross the outer membrane [21,22,23,24]. This protein is highly antigenic, containing both B and T-cell epitopes, and is currently the most promising vaccine antigen having completed a Phase I clinical trial [12,16,17,21,24,25,26,27,28,29,30,31,32].

Wang and Grayston [33,34,35] published findings based on protection studies in mice and serological analyses that grouped the 15 *C. trachomatis* serovars into three complexes C (C, J, H, I, A, K, L3), B (B, Ba, E, D, L1, L2) and G/F. They identified a senior to junior relationship within each complex such that the senior serovar, for example, C, protected against all the junior serovars, while the junior serovar, L3, protected only against itself. These findings have been supported in several studies, including in the genital challenge mouse model using recombinant MOMP [36]. Mice immunized with *C. trachomatis* serovar D MOMP were protected against shedding and infertility when challenged with serovars D or E, but not when challenged with serovar F [36]. To elicit protection against all 15 major serovars, a vaccine will require at least MOMP from the senior serovars of each of the three complexes. As an alternative, or in conjunction with MOMP, other more conserved chlamydial antigens may induce broad cross-serovar protection [17,37,38,39].

Humans and mice infected with *Chlamydia* mount an immune response to hundreds of proteins [40,41,42]. Using the *C. trachomatis* and *C. muridarum* mouse models, several investigators have reported that some of the more conserved proteins, such as the polymorphic membrane proteins (Pmp) and the plasmid glycoprotein 3 (Pgp3), can protect mice against genital and/or respiratory challenges [43,44,45,46,47,48].

*C. trachomatis* and *C. muridarum*, have nine *pmp* genes encoding Pmps (A, B, C, D, E, F, G, H, I) with MWs ranging from ~100–150 kDa [19,45,49,50,51,52]. Pmps have three functional domains: 1) a cleavable *sec*-dependent N-terminal signal for translocation through the cytoplasmic membrane, 2) a C-terminal β-barrel sequence for outer membrane insertion, and 3) a passenger domain for cell surface localization. Pmps are located on the chlamydial cell surface and have the capacity to adhere to the host cell [49,53,54,55,56].

In 2006, Crane et al. [53] reported in vitro assays whereby antibodies to PmpD neutralized all *C. trachomatis* serovars. Karunakaran et al. 2008 [57], using an immunoproteomic approach, discovered T-cell epitopes in four *C. muridarum* Pmps (E, F, G and H). Vaccination of C57BL/6, BALB/c, and C3H/HeN mice with the passenger domains from each of these four proteins (E, F, G and H), accelerated vaginal clearance of *C. muridarum*, while PmpG elicited the best overall protection [57].

In the intranasal challenge model, the passenger domains of the nine *C. trachomatis* serovar E Pmps, adjuvanted with CpG-1826 plus Montanide ISA 720 VG, were used to vaccinate BALB/c mice [58]. Based on disease burden and the number of *C. muridarum* IFU recovered from the lungs, mice immunized with *C. trachomatis* serovar E PmpC, were the best protected against a respiratory challenge. Limited protection was also observed in mice immunized with PmpG or H, suggesting that Pmps could elicit *C. trachomatis* cross-serovar and cross-species protection.

Most of the *C. trachomatis* isolates, and *C. muridarum,* have a plasmid that encodes for eight proteins including Pgp3 [59,60,61]. Pgp3 (MW 28 kDa) forms an ~84 kDa homotrimer and has been found in the membrane of *Chlamydia*, and in the cytoplasm of the host cells [62,63,64]. Antibodies from humans and mice bind to the homotrimer but not to the monomer [41,65]. Donati et al. [43] vaccinated C3H/HeN mice with a DNA plasmid expressing *C. trachomatis* serovar D Pgp3 and a control group with the same plasmid containing an irrelevant insert. Mice vaccinated with the *Pgp3* plasmid developed systemic and mucosal immune responses. As determined by the number of positive salpinx cultures, mice vaccinated with the *Pgp3* plasmid were partially protected against a vaginal challenge with serovar D. Intranasal vaccination of mice with a plasmid expressing *C. trachomatis* serovar D pORF5 (coding for Pgp3), were challenged vaginally with *C. muridarum* [66]. Following immunization, significant antigen-specific Th1 responses and antibody levels were detected. Bacterial shedding, length of time of shedding, and upper genital tract inflammation were reduced in the pORF5 immunized animals. Similar results were obtained by Luan et al. [44] using purified Pgp3 for vaccination.

In this study, we used *C. muridarum* recombinant MOMP, PmpG and Pgp3 proteins, alone and in combination, as vaccine antigens to establish their ability to induce humoral and cell-mediated immune responses, and to protect mice against a respiratory challenge with *C. muridarum*. The respiratory challenge route was used as a screening method to identify protective antigens that can then be tested in the genital challenge model. To induce both humoral and cell-mediated immune responses, CpG-1826, a TLR-9 agonist that elicits Th1 responses, and Montanide ISA 720 VG, a non-TLR agonist that stimulates Th2 responses, were used as adjuvants [25,67]. Our results show that mice vaccinated with MOMP mounted robust protective immune responses, while animals vaccinated with PmpG, or Pgp3, exhibited weaker immune responses. Furthermore, these two antigens in combination with MOMP, failed to enhance the protection induced by MOMP alone. Importantly, Pgp3 exerted an antagonistic effect on the protection elicited by MOMP, a finding that may explain its role as a *Chlamydia* virulence factor [68].

## 2. Methods and Methods

### 2.1. C. muridarum Stocks

The *C. muridarum* (MoPn; strain Nigg II; previously called *Chlamydia trachomatis* mouse pneumonitis) was purchased from the American Type Culture Collection (Manassas, VA, USA), and was grown in HeLa-229 cells. Elementary bodies (EB) were purified and stored at −80°C in sucrose phosphate–glutamate (SPG) buffer, as previously described [24].

### 2.2. Cloning, Expression and Purification of C. muridarum Proteins

*C. muridarum* EB, were used to isolate genomic DNA with the Wizard Genomic DNA Purification kit (Promega Corporation, Madison, WI, USA). *C. muridarum* DNA fragments used for cloning were obtained by PCR. MOMP was amplified, cloned, expressed and purified as previously described [26]. PCR-amplified DNA harboring *C. muridarum pgp3* and *PmpG* (TC263) genes were cloned into the NcoI-XhoI sites of the pET-45b(+) vector (Novagen, Madison, WI, USA) under the control of the T7 promoter, using the following primers (Integrated DNA Technologies Inc., Coralville, IA, USA): *Pgp3*-forward—5′-GCAGGTACCATGACAGAACCTCTTACAGATC-3′; *Pgp3*-reverse—5′-GCACTCGAGTTAAGTGTTTTTTTGAGGTATC-3′ (GenBank AAF39719.1). PmpG-forward—5′-GAGGGTACCATGGCTCGAATAGGTGGAGG-3′, PmpG-reverse—5′-GACCTCGAGTTAAGCTACGCGCTCCGGACCAGGA-3′ (GenBank AE002160). The *pgp3* DNA fragment codes for 240 amino acids and the *PmpG* for 410 amino acids, corresponding to the passenger domain of PmpG. The ligated vectors were used to transform *E. coli* DH5-alpha (New England BioLabs, Ipswich, MA, USA) competent cells. Resulting clones were selected and checked by PCR using DreamTaq^TM^ Green PCR Master Mix (Thermo Fisher Scientific, Waltham, MA, USA) for the appropriate insert size. To confirm the proper sequence of the insert and assure the in-frame cloning, selected clones were sequenced by GENEWIZ Inc., from Azenta Life Sciences (South Plainfield, NJ, USA).

*E. coli* BL21(DE3) (New England BioLabs) harboring either pET-45b(+)-*pgp3*, or pET-45b(+)-*pmpG*, were cultured at 37 °C with aeration and induced with 0.5 mM isopropyl-β-d-thiogalactopyranoside (IPTG) at 37 °C for 6 h. Lysozyme treatment and sonication were used to disrupt bacterial cells suspended in 20mM Na phosphate buffer [pH 7.4], containing 50 mM NaCl, 5% glycerol and 0.05% Z3-14 (Anatrace, Inc., Maumee, OH, USA) detergent followed by centrifugation at 20,000× *g* for 30 min at 4 °C. His-tagged Pgp3 and PmpG were purified using IMAC (Immobilized Metal Affinity Chromatography) over a His60 Ni Superflow^TM^ resin (Clontech Laboratories, Inc., Mountain View, CA, USA) column and gel filtration column with Sephacryl 300 [26].

Eluted proteins were purified using Pierce Endotoxin Removal Resin (Thermo Fisher Scientific). LPS was quantified using the Limulus Amebocyte Lysate Pyrotell assay (Associates of Cape Cod. Inc., East Falmouth, MA, USA). The antigens contained: 6xHis-Pgp3 protein (300 EU/mg/protein equal to 6.0 EU/20 μg/dose/mouse); 6xHis-PmpG (10 EU/mg/protein equal to 0.2 EU/20 mg/dose/mouse) and MOMP less than 0.5 EU/mg/protein. The purity and stability of the 6xHis proteins were assessed by polyacrylamide gel electrophoresis (PAGE). Final antigens preparations were concentrated and transferred into NaPi (20 mM), NaCl (50 mM), glycerol (5%), Z3-14 (0.05%), pH 7.4 buffer, and stored at −80°C until used for immunization.

### 2.3. Vaccination Protocols

Four to five-week-old female BALB/c (H-2^d^) mice (Charles River Laboratories; Wilmington, MA, USA) were housed at the University of California, Irvine, Vivarium. The University of California, Irvine IACUC approved all animal protocols.

The adjuvants CpG-1826 (TriLink, San Diego, CA, USA; 10 μg/mouse/immunization) and Montanide ISA 720 VG (SEPPIC Inc., Fairfield, NJ, USA; 70% of total vaccine volume) were directly mixed with single antigens (PmpG, Pgp3 or MOMP: 20 μg of each antigen/mouse/immunization) and antigens combinations (10 μg of each antigen/mouse/immunization).

Groups of 5 to 9 mice were immunized twice by the intramuscular (i.m.) route in the quadriceps muscle at a 4-week interval. An adjuvant control group was immunized with CpG-1826 and Montanide ISA 720 VG in phosphate buffered saline (PBS). Sera and vaginal washes were collected before immunization and the day before the challenge and were stored at −20 °C until use. To determine the cell-mediated immune responses, four mice per group were randomly selected and euthanized the day before the challenge. Four weeks after the last immunization mice were challenged intranasally (i.n.) with 10^4^ IFU of *C. muridarum*. All animal experiments were replicated once.

### 2.4. Determination of the Humoral Immune Responses Induced by Vaccination

To determine humoral responses, 96-multiwell plates were coated with *C. muridarum* EB (1 μg/well), or purified MOMP, Pgp3, or PmpG (0.1 μg/well), and incubated with serially diluted pre-immune sera, as a negative control, and sera collected the day before the challenge [69]. Horseradish peroxidase-conjugated goat anti-mouse IgG (KPL, diluted 1:5000), IgG1 and IgG2a (BD Pharmingen, diluted 1:2000) antibodies were added and the binding was measured in an EIA reader (Labsystem Multiscan, Helsinki, Finland). The geometric mean titers (GMTs) are expressed as the reciprocal of the dilution. In vaginal washes, the levels of *C. muridarum*-specific IgG and IgA (ICN Pharmaceutical, OH; diluted 1:3500) antibodies were determined using the same procedures.

In vitro neutralization assays were performed as described [70]. Two-fold serial dilutions of mouse sera, made with Ca^2+^- and Mg^2+^-free PBS, pH 7.2 and supplemented with 5% guinea pig serum, were added to 1 × 10^4^ IFU of *C. muridarum*. Following incubation for 45 min at 37 °C, the mixtures were inoculated by centrifugation into HeLa-229 cells grown on 96-multiwell plates. After 30 h of incubation at 37 °C, the monolayers were fixed and stained with a pool of monoclonal antibodies to *C. muridarum* generated in our lab. The titer of a sample was the dilution that yielded 50% neutralization relative to the negative control serum from PBS immunized mice.

Antibodies elicited by vaccination against linear epitopes of *C. muridarum* MOMP were determined using synthetic 25-mers overlapping peptides corresponding to the entire amino acid sequence of mature MOMP (SynBioSci Corp., Livermore, CA, USA). Peptide 25 overlapped the N-terminus and C-terminus of MOMP. Each peptide (1 μg/well) was adsorbed onto a high binding affinity 96-microttiter plate and antibody binding was assessed in triplicate using anti-mouse IgG.

### 2.5. Evaluation of C. muridarum—Specific Cellular Immune Responses Induced by Vaccination

Four mice per group were used to determine cellular immune responses. Animals were euthanized the day before the intranasal challenge and T-cell suspensions prepared from spleen cells purified using a nylon wool column as described [25]. T-cells were aliquoted into 96-well plates at a concentration of 2.5 × 10^6^ cells/well. The T-cells were stimulated with *C. muridarum* UV-inactivated EB, or purified MOMP. Concanavalin A (5 μg/mL) and culture media served as positive and negative controls, respectively. After two days of incubation at 37 °C, in a 5% CO_2_ incubator, supernatants were harvested and stored at −20 °C. Levels of IFN-γ and IL-4 in supernatants were determined by an ELISA (BD Pharmingen, San Diego, CA, USA) [25].

### 2.6. Intranasal Challenge and Evaluation of the Infection and Disease

Four weeks after the last immunization, anesthetized mice were challenged i.n. with 10^4^ IFU of *C. muridarum* [26]. The mice were weighed for 10 days, euthanized, their lungs weighed, homogenized in 5 mL of SPG (Seward Stomacher 80; Labsystems), and serial 10-fold dilutions were used to infect Hela-229 cell monolayers. The cultures were incubated for 30 h at 37 °C in a 5% CO_2_ incubator, the inclusions visualized with *C. muridarum*-specific monoclonal antibodies and counted using a light microscope. The limit of detection was, <50 *C. muridarum* IFU/lungs mouse [71].

To determine the local cellular immune responses, levels of IFN-γ in lungs’ supernatants at 10 days post-challenge (d.p.c.) were determined by an ELISA as describe above.

### 2.7. Statistical Analyses

Parametric and non-parametric statistical tests were used as follow. The Student’s *t*-test was employed to evaluate changes in body weight at day 10 p.c., lungs’ weights and amounts of IFN-γ in lungs supernatants. Repeated measures ANOVA was used to compare changes in mean body weight over the 10 days of observation following the *C. muridarum* i.n. challenge. The Mann–Whitney *U*-Test was used to compare antibodies titers, levels of IFN-γ and IL-4 in T-cell supernatants, and the number of *C. muridarum* IFU in the lungs. Values below the limit of detection (BLD) were assigned ½ the value of the BLD, as described by Beal [72]. A *p* value of < 0.05 was considered to be significant. A *p* value of <0.1 indicates approaching significance.

## 3. Results

### 3.1. Analyses of the Three C. muridarum Recombinant Antigens Used for Immunization

**Figure 1:**
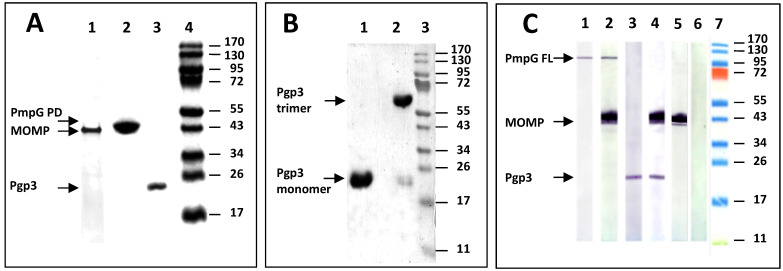
Characterization of the recombinant *C. muridarum* proteins used as antigens. (**A**): Silver-stained 10% SDS-PAGE of the *C. muridarum* recombinant proteins used as antigens. Lane 1: MOMP; Lane 2: PmpG (passenger domain); Lane 3: Pgp3 and Lane 4: MW protein standards. (**B**): Silver-stained 10% SDS-PAGE of the *C. muridarum* heated and non-heated Pgp3. Lane 1: Pgp3 was denatured by heat (10 min at 100 °C) before loading. Lane 2: Pgp3 was loaded on the gel without boiling. The gel was run overnight at 4 °C. (**C**): Detection by Western blot of MOMP, PmpG and Pgp3 in *C. muridarum* EB using serum from vaccinated mice. *C. muridarum* EBs were probed with sera from mice immunized with: Lane 1: PmpG; Lane 2: MOMP+PmpG; Lane 3: Pgp3; Lane 4: MOMP+Pgp3; Lane 5: MOMP; Lane 6: PBS, and Lane 7: MW protein standards.

Recombinantly produced *C. muridarum* protein antigens were used for this study. Using a sliver stain, the mature MOMP, and the passenger domain of PmpG, had similar MW (~40 kDa), while the full length Pgp3 had a MW of ~26 kDa (Figure 1A). Loading the samples, with and without boiling, in a 10% SDS-PAGE, both the denatured monomer and the non-denatured trimer forms of Pgp3 were detected by silver stain (Figure 1B). Using a Western blot, sera from mice vaccinated with a single, or two antigens, recognized their respective proteins in *C. muridarum* EB (Figure 1C).

### 3.2. Characterization of the Humoral Immune Responses Induced by Vaccination

**Figure 2:**
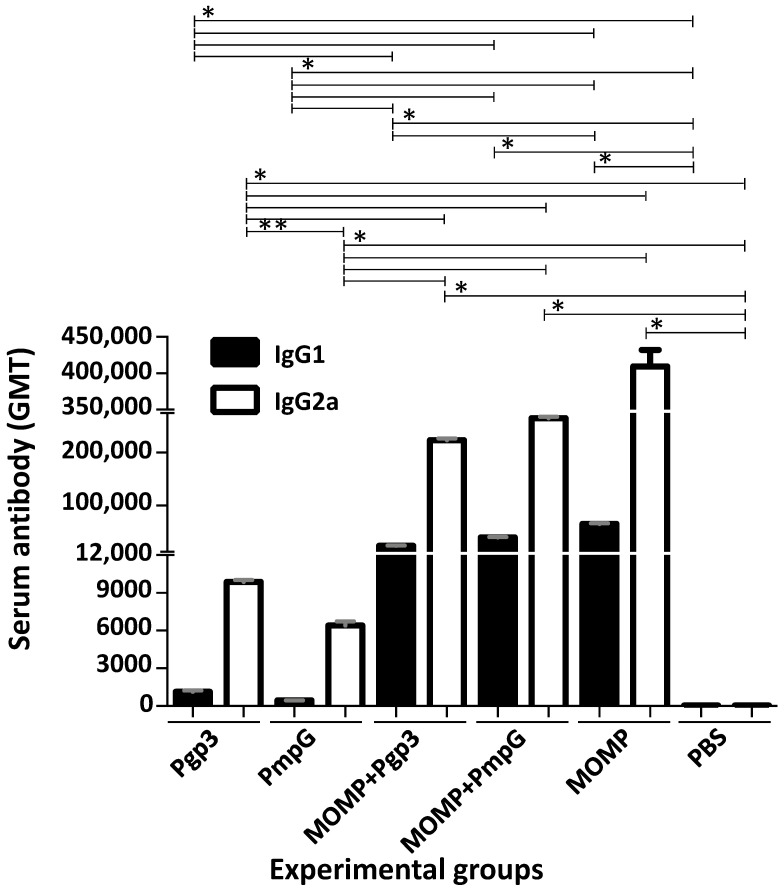
Vaccine-induced serum IgG1 and IgG2a antibody responses (GMT±SE) against *C. muridarum* EB the day before the i.n. challenge. Serum samples from immunized mice were collected the day before the i.n. challenge with *C. muridarum* and the titers of IgG2a and IgG1 determined to evaluate the Th1 versus Th2 humoral immune responses using EB as the antigen in an ELISA. BLD < 100. * *p* < 0.05 by the Mann–Whitney’s *U*-test. ** *p* < 0.1 by the Mann–Whitney’s *U*-test.

Following vaccination, humoral immune responses were determined the day before the i.n. challenge using *C. muridarum* EB as antigens (Figure 2 and Supplemental Table S1). MOMP vaccinated animals had an IgG2a antibody geometric mean titer (GMT) of 409,600 and an IgG1 GMT of 64,508. Mice immunized with Pgp3 had lower serum IgG2a (9870) and IgG1 (1131) GMT to *C. muridarum* EB. Similar levels of these antibodies were seen in mice vaccinated with PmpG (6400 and 436, respectively). High IgG2a antibody levels to EB were observed in animals vaccinated with MOMP+Pgp3 (223,336) or MOMP+PmpG (265,593). IgG1 GMT were also high for these two groups, 23,475 and 39,481, respectively. The IgG2a/IgG1 ratios ranged from 6.4 to 14.7, indicative of Th1-biased humoral immune responses in all vaccinated animals.

**Figure 3:**
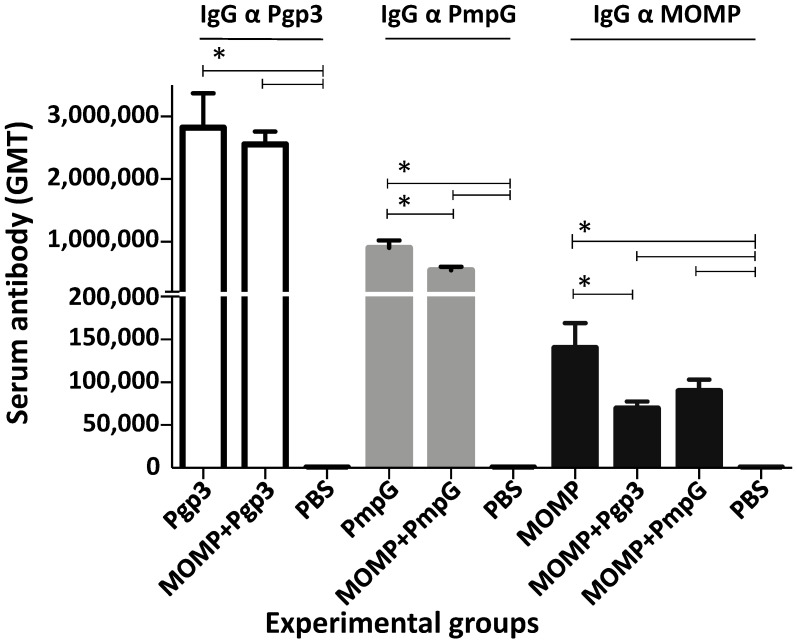
Vaccine-induced serum IgG antibody responses (GMT±SE) against MOMP, PmpG, or Pgp3 the day before the *C. muridarum* i.n. challenge. Blood was collected the day before the i.n. challenge and probed with MOMP, PmpG or Pgp3, individually or in combination, using an ELISA. BLD < 100. * *p* < 0.05 by the Mann–Whitney’s *U*-test.

Very high GMTs were detected when using the homologous protein as the antigen (Figure 3 and Supplemental Table S2). MOMP-vaccinated mice had an IgG titer of 139,900. The GMT to Pgp3 in mice immunized with this protein was 2,826,500 and for mice vaccinated with PmpG it was 905,100. When protein combinations were utilized to vaccinate animals, the antibody levels to the two respective homologous antigens were similar, or slightly lower, than when the individual protein was utilized for immunization. For example, mice vaccinated with MOMP+PmpG had a GMT of 89,800 to MOMP and a GMT of 543,500 to PmpG.

**Figure 4:**
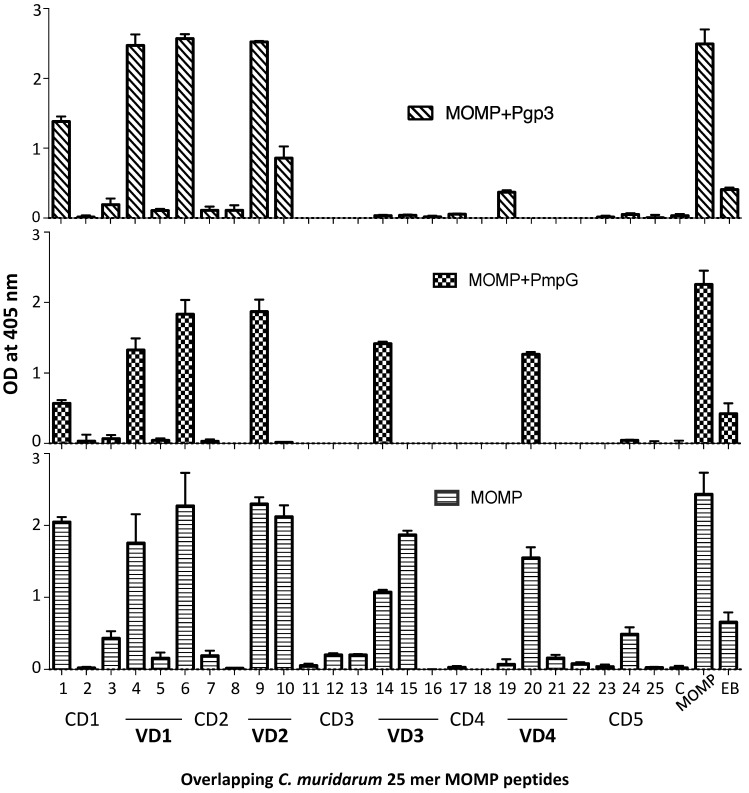
Detection of serum antibodies specific for *C. muridarum* MOMP peptides (OD_405_ ± SD). Serum samples from immunized mice were collected the day before the i.n. challenge and their reactivity to 25-mer overlapping peptides corresponding to the whole amino acid sequence of mature *C. muridarum* MOMP were analyzed by ELISA. MOMP and *C. muridarum* EB were used as positive controls.

Epitope mapping, using MOMP synthetic peptides, demonstrated that mice vaccinated with MOMP produced antibodies to the four VDs (Figure 4). Peptides in the first constant domain (CD1) were also recognized. Animals immunized with MOMP+PmpG showed a similar pattern of antibody specificity. In contrast, the group of mice immunized with MOMP+Pgp3 failed to generate significant amounts of antibodies to VD3 and weak to VD4.

**Figure 5:**
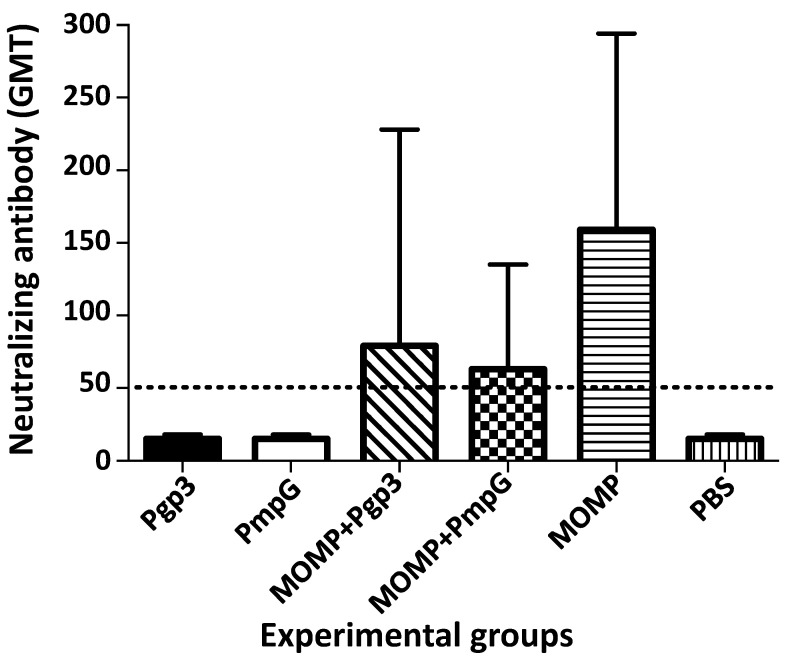
Vaccine elicited levels of neutralizing antibody (GMT±SE) in sera the day before the *C. muridarum* i. n. challenge. Serum samples were incubated with *C. muridarum* live EBs and the neutralization titer was determined by the dilution of the sera that decreased the number of *C. muridarum* IFU by 50% compared with the PBS immunized group. BLD (horizontal broken line) < 50.

Neutralizing antibodies were determined in serum samples using live EB as the antigen (Figure 5 and Supplemental Table S1). Only the three groups of mice vaccinated with MOMP alone (159), or in combination with PmpG (63), or Pgp3 (79), had neutralizing antibodies, indicating that they were elicited by MOMP. No significant differences in neutralizing titers were observed between these three groups of mice. Immunization with PmpG, or Pgp3 did not induce neutralizing antibodies.

**Figure 6:**
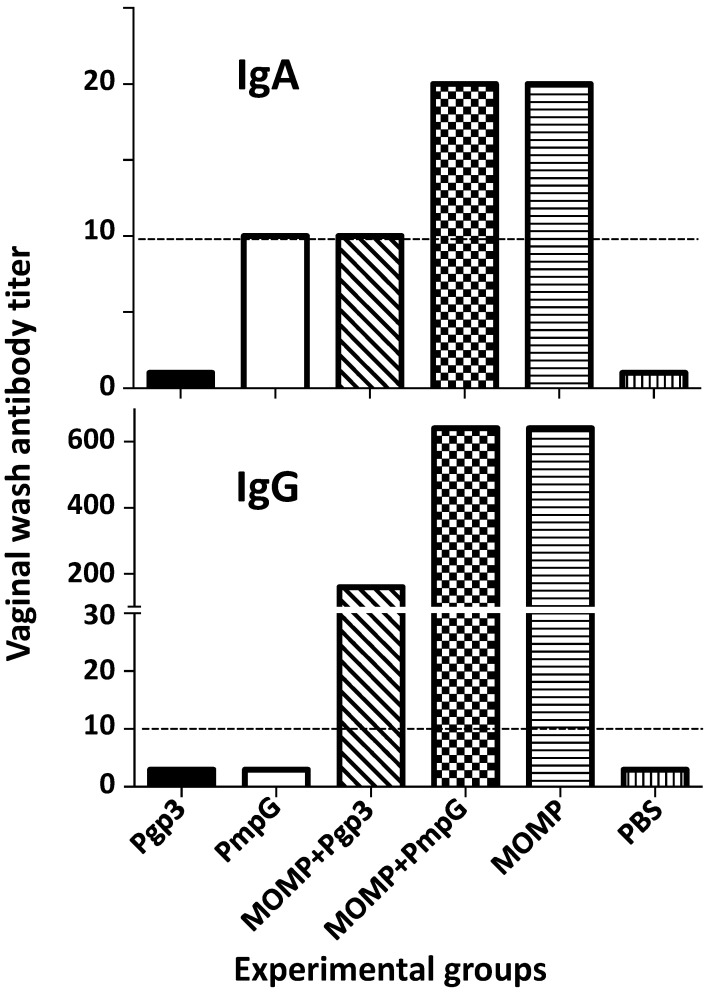
*C. muridarum*-specific IgA and IgG (GMT) in vaginal washes from vaccinated mice the day before the i.n. challenge. Vaginal washes were collected and pooled from each group of mice and the *C. muridarum* specific IgA and IgG titer determined using EB as the antigen in an ELISA. BLD (horizontal broken line) < 10.

IgG and IgA antibody levels to *C. muridarum* EB were determined in pooled vaginal washes. As shown in Figure 6 and Supplemental Table S3, only mice immunized with MOMP alone, or in combination with Pgp3 or PmpG, showed IgG in the vaginal washes. IgA levels were negative or very low in all groups of mice.

### 3.3. Cell-Mediated Immune Responses following Vaccination

**Figure 7:**
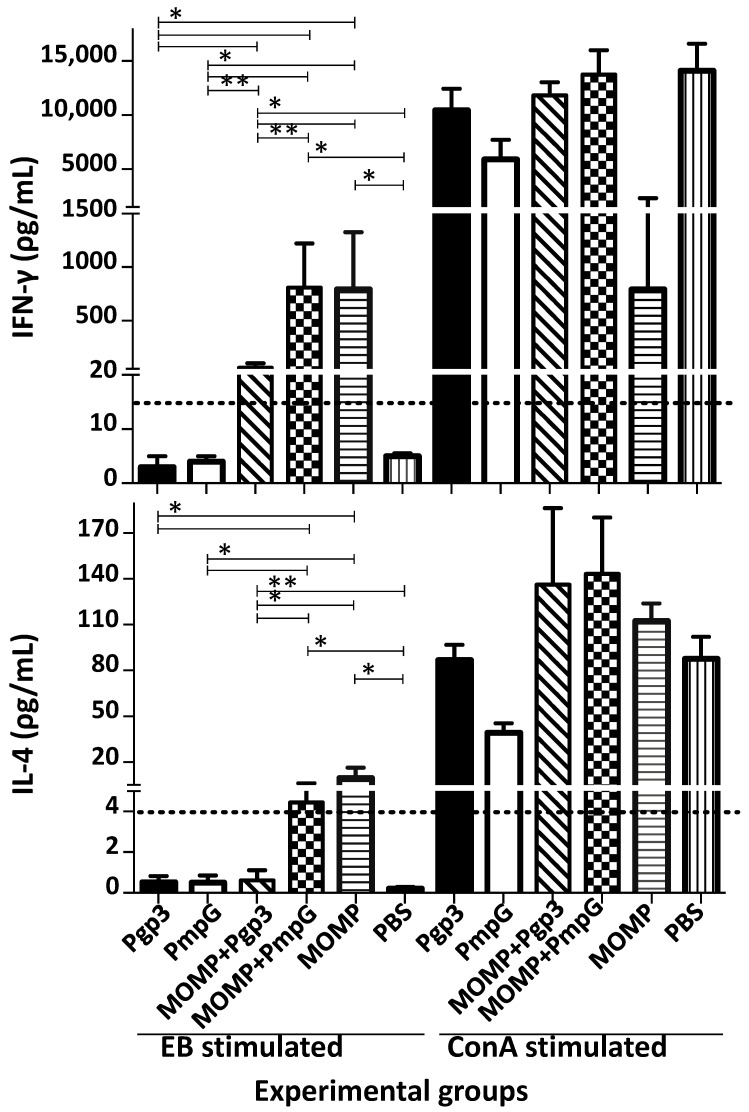
Evaluation of the cytokine responses from T-cells isolated from the spleens of vaccinated mice the day before the i.n. challenge. Spleens from four mice from each immunized group were collected the day before the i.n. challenge, T-cells separated with nylon wool and stimulated with *C. muridarum* EB, and ConA as a positive control. Levels of IFN-γ (pg/mL ± SE) and IL-4 (pg/mL ± SE) were determined as indicators of Th1 and Th2 responses, respectively. * *p* < 0.05 by the Mann–Whitney’s *U*-test. ** *p* < 0.1 by the Mann–Whitney’s *U*-test. BLD (horizontal broken line) IFN-γ < 15 pg/mL; IL-4 < 4 pg/mL.

The day before the intranasal challenge, four mice per group were euthanized, their spleens collected, and T-cells separated using nylon wool. T-cells from mice immunized with MOMP, and stimulated with EB, secreted 796.05 pg/mL of IFN-γ (Figure 7 and Supplemental Table S4). T-cells from mice immunized with Ppg3 or PmpG, stimulated with EB, did not secrete significant amounts of IFN-γ (<15 pg/mL). In contrast, T-cells from mice immunized with MOMP in combination with Ppg3 or PmpG, stimulated with EB, secreted significant levels of IFN-γ, 60.70 and 811.38 pg/mL, respectively. No significant differences were observed in amounts of IFN-γ produced between mice immunized with MOMP alone or with MOMP+PmpG (*p* > 0.05); however, the group immunized with MOMP+Pgp3 secreted lower IFN-γ levels (*p* < 0.05). Levels of IL-4 were low, or negative, in all groups. When T-cells were stimulated with ConA, as a positive control, levels of IFN-γ and of IL-4 were significant indicating that the T-cells were viable.

### 3.4. Changes in Body Weight following the Intranasal Challenge

**Figure 8:**
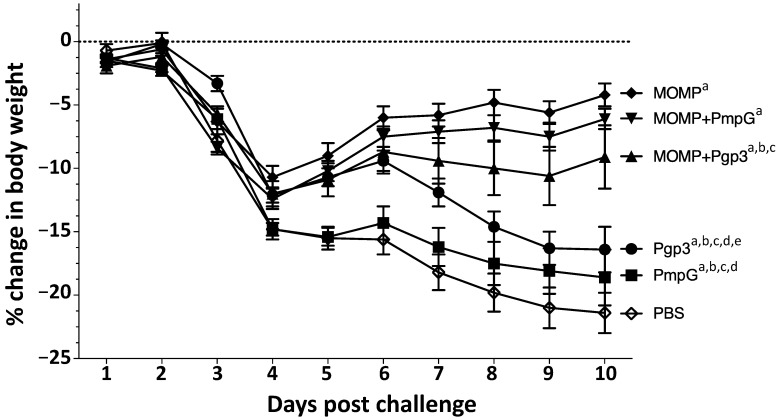
Body weight loss following the i.n. challenge with *C. muridarum*. Mice were immunized with several vaccine formulations and challenged i.n. with 10^4^ *C. muridarum* IFU at four weeks after the last immunization. Daily percentage changes in mean body weight following the i.n. challenge. ^a^ *p* < 0.05 by the repeated-measures ANOVA compared with the PBS immunized group. ^b^ *p* < 0.05 by the repeated-measures ANOVA compared with the MOMP immunized group. ^c^ *p* < 0.05 by the repeated-measures ANOVA compared with the MOMP+PmpG immunized group. ^d^ *p* < 0.05 by the repeated-measures ANOVA compared with the MOMP+Pgp3 immunized group. ^e^ *p* < 0.05 by the repeated-measures ANOVA compared with the PmpG immunized group.

Four weeks after the last immunization, mice were challenged intranasally with 10^4^ *C. muridarum* IFU. Body weight changes were used as a parameter indicative of the systemic effects of the infection. All groups of mice rapidly lost body weight from day 2 to day 4 post challenge (p.c.) (Figure 8).

The negative control receiving PBS with adjuvants continuously lost body weight for the entire 10 days of observation. Mice vaccinated with MOMP, gained weight starting at day 5 p.c. Mice immunized with Pgp3 gained body weight from day 4 to day 6 p.c. but then lost body weight, while the group vaccinated with PmpG lost body weight for most of the 10 days p.c. In contrast, mice vaccinated with MOMP+PmpG, gained weight from day 4 to day 10, while those immunized with MOMP+Pgp3 gained body weight from day 4 to day 6 p.c., but then lost weight until the end of the experiment. Using the repeated-measures ANOVA to calculate differences in body-weight losses over the 10 p.c. days of observation, the weight loss of all immunized mice was significantly lower than the negative control receiving PBS with adjuvants (*p* < 0.05). The two groups of mice that best maintained their body weight were those immunized with MOMP, and MOMP+PmpG. Mice vaccinated with Pgp3, or PmpG alone, lost more body weight than their respective combination groups immunized with MOMP (*p* < 0.05).

**Figure 9:**
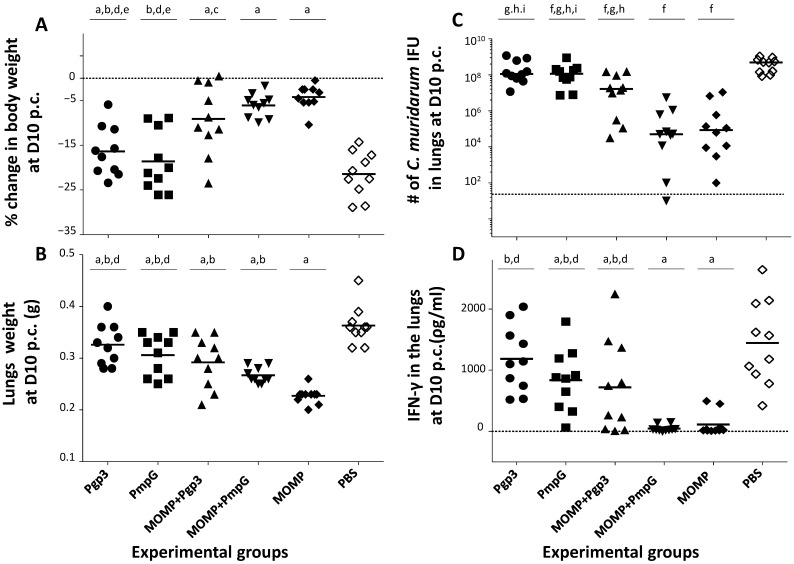
Systemic and local disease burden and local immune responses in the lungs in vaccinated mice following the i.n. challenge with *C. muridarum*. (**A**) Percentage change in mean body weight at D10 following the i.n. challenge. The mean is shown as a horizontal line. Each symbol represents a single animal. ^a^ *p* < 0.05 by the Student’s *t*-test compared with the PBS immunized group. ^b^ *p* < 0.05 by the Student’s *t*-test compared with the MOMP immunized group. ^c^ *p* < 0.1 by the Student’s *t*-test compared with the MOMP immunized group. ^d^ *p* < 0.05 by the Student’s *t*-test compared with the MOMP+PmpG immunized group. ^e^ *p* < 0.05 by the Student’s *t*-test compared with the MOMP+Pgp3 immunized group. (**B**) Lungs weight (g) at D10 after the i.n. challenge. The mean is shown as a horizontal line. Each symbol represents a single animal. ^a^ *p* < 0.05 by the Student’s *t*-test compared with the PBS immunized group. ^b^ *p* < 0.05 by the Student’s *t*-test compared with the MOMP immunized group. ^d^ *p* < 0.05 by the Student’s *t*-test compared with the MOMP+PmpG immunized group. (**C**) Number of *C. muridarum* IFU recovered from the lungs at D10 after the i.n. challenge. The median is shown as a horizontal line. Each symbol represents a single animal. ^f^ *p* < 0.05 by the Mann–Whitney’s *U*-test compared with the PBS immunized group. ^g^ *p* < 0.05 by the Mann–Whitney’s *U*-test compared with the MOMP immunized group. ^h^ *p* < 0.05 by the Mann–Whitney’s *U*-test compared with the MOMP+PmpG immunized group. ^i^ *p* < 0.05 by the Mann–Whitney’s *U*-test compared with the MOMP+Pgp3 immunized group. BLD (horizontal broken line) < 50 *C. muridarum* IFU. (**D**) Levels of IFN-γ (pg/mL) detected in the lungs at D10 after the i.n. challenge. The mean is shown as a horizontal line. Each symbol represents a single animal. ^a^ *p* < 0.05 by the Student’s *t*-test compared with the PBS immunized group. ^b^ *p* < 0.05 by the Student’s *t*-test compared with the MOMP immunized group. ^d^ *p* < 0.05 by the Student’s *t*-test compared with the MOMP+PmpG immunized group. BLD (horizontal broken line) < 15 pg/mL.

By day 10 p.c., in comparison with the PBS control animals (body-weight loss 21.4%), the five groups of mice immunized with one or two chlamydial antigens were protected (*p* < 0.05) (Figure 9A and Table 1). The body-weight losses of mice vaccinated only with Pgp3 (16.4%), or PmpG (18.6%), or with the combination of MOMP+Pgp3 (9.1%), were significant when compared with MOMP alone (4.2%) vaccinated animals (*p* < 0.05). Only mice immunized with MOMP+PmpG (6.1%) had a body-weight loss that was not significantly different when compared to the MOMP only vaccinated group (*p* > 0.05).

### 3.5. Lung’s Weights

As a measure of the local inflammatory responses, the weights (g) of the mice lungs were determined at D10 p.c. (Figure 9B and Table 1). The lung weights of all groups immunized with a chlamydial antigen were significantly different from the negative control receiving PBS plus the adjuvants (*p* < 0.05). The mean lung weights of mice vaccinated with MOMP was 0.23 g and 0.36 g for those receiving PBS (*p* < 0.05). The lung weights of mice immunized with Pgp3 (0.33 g), or PmpG (0.31 g) alone, or in combination with MOMP (0.29 g and 0.27 g, respectively), were significantly different from the controls immunized with MOMP (*p* < 0.05). Animals immunized with MOMP+PmpG had significantly lower lung weights than those vaccinated with PmpG alone (*p* < 0.05). No significant differences were observed between the lung weights of mice vaccinated with Pgp3 or PmpG (*p* > 0.05).

### 3.6. Burden of C. muridarum in the Lungs

At D10 p.c., the median number of *C. muridarum* IFU recovered from the lungs of mice vaccinated with MOMP was 0.09 × 10^6^, while in the negative control group receiving PBS plus the adjuvants, it was 498.30 × 10^6^ (*p* < 0.05) (Figure 9C and Table 1). In comparison with the negative control group, except animals immunized with Pgp3 (114.76 × 10^6^), all mice vaccinated with a chlamydial antigen were protected (*p* < 0.05). Mice immunized with MOMP+PmpG (0.05 × 10^6^) showed a similar level of protection as those vaccinated with MOMP alone (*p* > 0.05). Mice immunized with MOMP+PmpG were also better protected than those vaccinated with MOMP+Pgp3 (16.50 × 10^6^) (*p* < 0.05).

### 3.7. Local Immune Responses in the Lungs

To evaluate local immune parameters that correlate with protection, levels of IFN-γ were determined in supernatants from lungs harvested at D10 p.c. (Figure 9D and Table 1). We expect that protected mice will have controlled the *C. muridarum* infection and, therefore, have low amounts of IFN-γ in lungs supernatants. Levels of IFN-γ (pg/mL) in lungs supernatants of mice immunized with MOMP (112.5) were significantly lower from those receiving PBS (1444.4) (*p* < 0.05). No significant differences in the levels of IFN-γ in the lungs were determined when comparing mice vaccinated with MOMP only versus PmpG+MOMP immunized mice (46.0) (*p* > 0.05). Amounts of IFN-γ in mice vaccinated with MOMP, were statistically significantly lower than in animals immunized with Pgp3 (1184.8) or PmpG (836.2) only, or with the MOMP+Pgp3 (719.8) combination, indicative that in these three groups the infection was still active (*p* < 0.05).

## 4. Discussion

The goal of this study was to determine the ability of MOMP, PmpG and Pgp3 antigens, alone and in combination, to induce protective immune responses against a *C. muridarum* intranasal challenge. As determined by changes in body weight, weight of the lungs and number of *Chlamydia* IFU recovered from the lungs, vaccines formulated with MOMP elicited robust protective immune responses while those induced by PmpG, or Pgp3 were weak. Combining MOMP with each of these two antigens elicited significant humoral and cellular immune responses that were protective. However, MOMP alone induced more robust protective immune responses than its combination with PmpG or Pgp3. Pgp3 had an antagonistic effect that significantly decreased the humoral and cell mediated protective immune responses elicited by MOMP.

MOMP, PmpG and Pgp3 have been shown to elicit protective immune responses in mice against genital and/or respiratory challenges [17,73,74,75,76,77]. Here, we wanted to determine if PmpG and/or Pgp3, well-conserved proteins among all the *C. trachomatis* serovars, could broaden the protection induced by MOMP, an antigen that only induces serovar/serogroup protection. Mice vaccinated with MOMP developed high antibody titers to *C. muridarum* EB while those immunized with PmpG, or Pgp3, mounted low antibody responses. Similar results were observed when levels of IgG and IgA were determined in vaginal washes using EB as the antigen, and when T-cell mediated immune responses were evaluated. These differences likely represent the relative quantities and accessibility of these three proteins in EB [78]. This possibility was confirmed when the antibody responses were determined against the purified proteins. In this case, both PmpG and Pgp3 immunized mice had high antibody titers against the homologous protein, confirming previous results in humans [40,79]. However, only vaccination with MOMP elicited neutralizing antibodies in serum. This is not surprising, since it is known that human and murine antibodies against the Pgp3 trimer are not neutralizing and we are not aware of any publication showing that PmpG elicits neutralizing antibodies [63,65,80]. Cell-mediated immune response, using EB as the stimulating antigen, followed a similar pattern. The stronger humoral and cellular immune responses to EB, elicited by MOMP, correlated with protection against the respiratory challenge. While mice vaccinated with MOMP mounted robust protection against body-weight losses, inflammatory responses in the lungs and number of *C. muridarum* IFU in the lungs, those immunized with PmpG, or Pgp3, were weakly protected.

Using multivalent vaccines, synergistic, additive, neutral or antagonistic effects may be observed [81,82,83,84,85]. For example, Finco et al. [83] identified *C. muridarum* antigens that elicited both humoral and cell-mediated immune responses. Mice immunized with each of these four antigens, TC0106, TC0210, TC0313, or TC0741 (10 μg of each antigen/dose), adjuvanted with LTK63+CpG, had 0.5–0.9 log_10_ reduction in the number of IFU recovered from the lungs. A four-antigen combination (TC0106, TC0210, TC0313 and TC0741; 10 μg of each antigen/dose) was then used to immunize mice. This multivalent combination resulted in a 4.1 log_10_ reduction in the number of *C. muridarum* IFU recovered from the lungs, indicative of synergistic effects. Yu et al. [82] also found additive effects of three *C. muridarum* antigens PmpE/F, PmpG, and MOMP in the genital model (single antigen, 5 μg/dose; three antigens, 1.67 μg/each per dose). As determined by vaginal shedding, the combination of the three proteins, adjuvanted with CAF01, exhibited the highest level of protection against a genital challenge. Coler et al. [84] also showed that MOMP, combined with CT875, (10 μg of each antigen, alone or in combination/dose), and adjuvanted with AS01B, elicited better protection against a vaginal challenge with *C. trachomatis* serovar K (UW-31/Cx), than the individual antigens.

Neutral effects resulting from immunization with *C. muridarum* antigens combinations have also been reported. For example, Cheng et al. [86] vaccinated mice with components of the *C. muridarum* putative ATP synthase complex TC0580, TC0581, TC0582, TC0584, or only with MOMP (10 μg of each antigen/dose). In addition, TC0582 was formulated in combination with TC0580, TC0581 or MOMP (10 μg of each antigen/dose). Animals immunized with combinations of two of these three antigens were only protected as well as mice vaccinated with MOMP, the most protective protein in the formulations. Li et al. [87] reported that the addition of CPAF to MOMP, or IncA, (15 μg of protein/dose), from *C. trachomatis* serovar D (UW3-Cx), did not enhance the CPAF (15 μg of protein/dose) induced protective effects on *C. muridarum* clearance, or oviduct pathology.

Here, we observed a neutral effect in immune responses and protection when combining MOMP with PmpG, while the formulation of MOMP with Pgp3 induced an antagonist effect. Qiu et al. have also described an antagonist effect when testing HCV antigens [85]. Although a group of mice vaccinated with a 10 μg/dose of MOMP, was not included, we have performed several experiments in mice immunized with 10 μg of MOMP and saw similar levels of protection as that observed here with 20 μg of MOMP [58,88]. Therefore, we favor the interpretation that PmpG had a neutral effect, while Pgp3 had an antagonistic activity when combined with MOMP.

Analyses of the immune responses support this premise. Antibody responses in serum and vaginal washes against EB, or MOMP, declined in mice immunized with MOMP+PmpG, or MOMP+Pgp3, when compared with those vaccinated only with MOMP. Neutralizing antibodies in serum, were also lower in mice vaccinated with the antigens combinations. These differences in humoral immune responses, for the most part, however, were not statistically different. Epitope mapping, using MOMP peptides, showed marked decreases against VD3 and VD4 in mice immunized with MOMP+Pgp3 versus those vaccinated with MOMP alone. The most striking differences, however, were observed in the cellular immune responses. Mice immunized with MOMP+PmpG had similar IFN-γ levels, in T-cell supernatants stimulated with EB, compared to those vaccinated with MOMP alone (811.38 versus 796.05 pg/mL; *p* > 0.05). In contrast, levels of IFN-γ from mice vaccinated with MOMP, were significantly higher than in mice immunized with MOMP plus Pgp3 (796.05 versus 60.70 pg/mL; *p* < 0.05).

Liu et al. [68] demonstrated, in the mouse model, that *C. muridarum* Pgp3 is a virulence factor. Chen et al. [63] postulated that the high antibody titers to Pgp3, present in *Chlamydia* infected humans and mice, may be exhausting the host humoral immune responses and, thus, providing an immune escape mechanism for this pathogen. These authors also reported the presence of Pgp3 trimers in the chlamydial outer membrane complex (COMC) where MOMP is the predominant component [63]. Although we do not have data about the interactions that occurred in the vaccine formulation between MOMP and Pgp3, and the mechanisms involved in the immune suppression, based on these findings, we hypothesize that the virulence of Pgp3 could be due to its interference with the protective humoral and cell-mediated immune responses elicited by MOMP.

A limitation of this study is the need to verify in a mouse model the interaction between MOMP, and Pgp3, observed here. Mutant *C. muridarum* constructs not expressing Pgp3, or monoclonal antibodies to Pgp3, could be utilized to specifically address this issue [89]. It may also be important to verify if Pgp3 has a similar effect on other potential vaccine antigens. When formulating a multivalent subunit chlamydial vaccine, it will be necessary to carefully evaluate the interactions between the antigens in animal models before implementation in humans.

To conclude, these results show that neither PmpG, or Pgp3, alone or in combination with MOMP, elicit better protection than MOMP alone against a respiratory challenge and, therefore, we expect similar outcomes in the genital challenge model. To protect against all the *C. trachomatis* serovars, the search for a well-conserved antigen that elicits broad protective immune responses should continue. In the meantime, the use of the three MOMPs from the senior serovars of each of the three complexes (J, E and G), appears to be the best option for the formulation of a broadly protective *C. trachomatis* vaccine.

## Figures and Tables

**Table 1:** Disease burden, yields of *C. muridarum* IFU, and IFN-γ in the lung’s supernatants at D10 post challenge.

Immunization Groups	% Change Body Weight (Mean ± 1 SE)	Lungs Weight (g) (Mean ± 1 SE)	Median Number IFU Recovered from Lungs (Min–Max) × 10^6^	IFN-γ (pg/mL) (Mean ± 1 SE)
Pgp3	−16.4 ± 1.8 ^a,b,d,e^	0.33 ± 0.01 ^a,b,d^	114.76 (11.78–1177.80) ^g,h,i^	1184.8 ± 170.6 ^b,d^
PmpG	−18.6 ± 2.2 ^b,d,e^	0.31 ± 0.01 ^a,b,d^	115.89 (7.40–906.00) ^f,g,h,i^	836.2 ± 161.0 ^a,b,d^
MOMP + Pgp3	−9.1 ± 2.5 ^a,c^	0.29 ± 0.02 ^a,b^	16.50 (0.03–154.02) ^f,g,h^	719.8 ± 241.0 ^a,b,d^
MOMP + PmpG	−6.1 ± 0.8 ^a^	0.27 ± 0.00 ^a,b^	0.05 (BLD–5.44) ^f^	46.0 ± 16.9 ^a^
MOMP	−4.2 ± 0.9 ^a^	0.23 ± 0.00 ^a^	0.09 (BLD–10.87) ^f^	112.5 ± 60.3 ^a^
PBS	−21.4 ± 1.6	0.36 ± 0.01	498.30 (84.56–1087.20)	1444.4 ± 219.6

^a^*p* < 0.05 by the Student’s *t*-test compared with the PBS + adjuvants immunized group. ^b^ *p* < 0.05 by the Student’s *t*-test compared with the MOMP immunized group. ^c^ *p* < 0.1 by the Student’s *t*-test compared with the MOMP immunized group. ^d^ *p* < 0.05 by the Student’s *t*-test compared with the MOMP + PmpG immunized group. ^e^ *p* < 0.05 by the Student’s *t*-test compared with the MOMP + Pgp3 immunized group. ^f^ *p* < 0.05 by the Mann-Whitney’s *U*-test compared with the PBS + adjuvants immunized group. ^g^ *p* < 0.05 by the Mann-Whitney’s *U*-test compared with the MOMP immunized group. ^h^ *p* < 0.05 by the Mann-Whitney’s *U*-test compared with the MOMP + PmpG immunized group. ^i^ *p* < 0.05 by the Mann-Whitney’s *U*-test compared with the MOMP + Pgp3 immunized group. BLD: Below level of detection (<50 *C. muridarum* IFU/lungs mouse).

## Data Availability

The datasets generated/analyzed during the current study are available from the corresponding author upon reasonable request.

## Supplementary Materials

The following supporting information can be downloaded at: https://www.mdpi.com/article/10.3390/vaccines11030504/s1, Table S1: Vaccine induced antibody responses in sera the day before the *C. muridarum* intranasal challenge; Table S2: Vaccine induced antibody responses in sera the day before the *C. muridarum* intranasal challenge; Table S3: *C. muridarum*-specific IgG and IgA titers in vaginal washes the day before the *C. muridarum* i.n. challenge; Table S4: In vitro cytokine production by T cells from immunized mice the day before challenge.

## References

[1] CDC (2021). Sexually transmitted disease surveillance 2019. Prevention DoS.

[2] Darville T. (2013). Recognition and treatment of chlamydial infections from birth to adolescence. Adv. Exp. Med. Biol..

[3] Adachi K.N., Nielsen-Saines K., Klausner J.D. (2021). Chlamydia trachomatis Screening and Treatment in Pregnancy to Reduce Adverse Pregnancy and Neonatal Outcomes: A Review. Front. Public Health.

[4] Gotz H., Lindback J., Ripa T., Arneborn M., Ramsted K., Ekdahl K. (2002). Is the increase in notifications of Chlamydia trachomatis infections in Sweden the result of changes in prevalence, sampling frequency or diagnostic methods?. Scand. J. Infect. Dis..

[5] Rekart M.L., Gilbert M., Meza R., Kim P.H., Chang M., Money D.M., Brunham R.C. (2013). Chlamydia public health programs and the epidemiology of pelvic inflammatory disease and ectopic pregnancy. J. Infect. Dis..

[6] Chesson H.W., Spicknall I.H., Bingham A., Brisson M., Eppink S.T., Farnham P.G., Kreisel K.M., Kumar S., Laprise J.F., Peterman T.A. (2021). The Estimated Direct Lifetime Medical Costs of Sexually Transmitted Infections Acquired in the United States in 2018. Sex. Transm. Dis..

[7] Schachter J., Dawson C.R. (1978). Human Chlamydial Infections.

[8] Nichols R.L., Bell S.D., Murray E.S., Haddad N.A., Bobb A.A. (1966). Studies on trachoma. V. Clinical observations in a field trial of bivalent trachoma vaccine at three dosage levels in Saudi Arabia. Am. J. Trop. Med. Hyg..

[9] Taylor H.R. (2008). Trachoma: A Blinding Scourge from the Bronze Age to the Twenty-First Century.

[10] Wang S.P., Grayston J.T., Alexander E.R. (1967). Trachoma vaccine studies in monkeys. Am. J. Ophthalmol..

[11] Woolridge R.L., Grayston J.T., Chang I.H., Cheng K.H., Yang C.Y., Neave C. (1967). Field trial of a monovalent and of a bivalent mineral oil adjuvant trachoma vaccine in Taiwan school children. Am. J. Ophthalmol..

[12] Farris C.M., Morrison R.P. (2011). Vaccination against Chlamydia Genital Infection Utilizing the Murine C. muridarum Model. Infect. Immun..

[13] Brunham R.C., Rey-Ladino J. (2005). Immunology of Chlamydia infection: Implications for a Chlamydia trachomatis vaccine. Nat. Rev. Immunol..

[14] Morrison R.P., Lyng K., Caldwell H.D. (1989). Chlamydial disease pathogenesis. Ocular hypersensitivity elicited by a genus-specific 57-kD protein. J. Exp. Med..

[15] Rockey D.D., Wang J., Lei L., Zhong G. (2009). Chlamydia vaccine candidates and tools for chlamydial antigen discovery. Expert Rev. Vaccines.

[16] Abraham S., Juel H.B., Bang P., Cheeseman H.M., Dohn R.B., Cole T., Kristiansen M.P., Korsholm K.S., Lewis D., Olsen A.W. (2019). Safety and immunogenicity of the chlamydia vaccine candidate CTH522 adjuvanted with CAF01 liposomes or aluminium hydroxide: A first-in-human, randomised, double-blind, placebo-controlled, phase 1 trial. Lancet Infect. Dis..

[17] de la Maza L.M., Darville T.L., Pal S. (2021). Chlamydia trachomatis vaccines for genital infections: Where are we and how far is there to go?. Expert Rev. Vaccines.

[18] Fitch W.M., Peterson E.M., de la Maza L.M. (1993). Phylogenetic analysis of the outer-membrane-protein genes of Chlamydiae, and its implication for vaccine development. Mol. Biol. Evol..

[19] Stephens R.S., Kalman S., Lammel C., Fan J., Marathe R., Aravind L., Mitchell W., Olinger L., Tatusov R.L., Zhao Q. (1998). Genome sequence of an obligate intracellular pathogen of humans: Chlamydia trachomatis. Science.

[20] Stephens R.S., Sanchez-Pescador R., Wagar E.A., Inouye C., Urdea M.S. (1987). Diversity of Chlamydia trachomatis major outer membrane protein genes. J. Bacteriol..

[21] Sun G., Pal S., Sarcon A.K., Kim S., Sugawara E., Nikaido H., Cocco M.J., Peterson E.M., de la Maza L.M. (2007). Structural and functional analyses of the major outer membrane protein of Chlamydia trachomatis. J. Bacteriol..

[22] Feher V.A., Randall A., Baldi P., Bush R.M., de la Maza L.M., Amaro R.E. (2013). A 3-dimensional trimeric beta-barrel model for Chlamydia MOMP contains conserved and novel elements of Gram-negative bacterial porins. PLoS ONE.

[23] Caldwell H.D., Perry L.J. (1982). Neutralization of Chlamydia trachomatis infectivity with antibodies to the major outer membrane protein. Infect. Immun..

[24] Caldwell H.D., Kromhout J., Schachter J. (1981). Purification and partial characterization of the major outer membrane protein of Chlamydia trachomatis. Infect. Immun..

[25] Pal S., Peterson E.M., de la Maza L.M. (2005). Vaccination with the Chlamydia trachomatis major outer membrane protein can elicit an immune response as protective as that resulting from inoculation with live bacteria. Infect. Immun..

[26] Sun G., Pal S., Weiland J., Peterson E.M., de la Maza L.M. (2009). Protection against an intranasal challenge by vaccines formulated with native and recombinant preparations of the Chlamydia trachomatis major outer membrane protein. Vaccine.

[27] Kari L., Whitmire W.M., Crane D.D., Reveneau N., Carlson J.H., Goheen M.M., Peterson E.M., Pal S., de la Maza L.M., Caldwell H.D. (2009). Chlamydia trachomatis native major outer membrane protein induces partial protection in nonhuman primates: Implication for a trachoma transmission-blocking vaccine. J. Immunol..

[28] Tifrea D.F., Pal S., Popot J.L., Cocco M.J., de la Maza L.M. (2014). Increased immunoaccessibility of MOMP epitopes in a vaccine formulated with amphipols may account for the very robust protection elicited against a vaginal challenge with Chlamydia muridarum. J. Immunol..

[29] Teng A., Cruz-Fisher M.I., Cheng C., Pal S., Sun G., Ralli-Jain P., Molina D.M., Felgner P.L., Liang X., de la Maza L.M. (2012). Proteomic identification of immunodominant chlamydial antigens in a mouse model. J. Proteom..

[30] Caldwell H.D., Schachter J. (1982). Antigenic analysis of the major outer membrane protein of *Chlamydia* spp.. Infect. Immun..

[31] O’Meara C.P., Armitage C.W., Kollipara A., Andrew D.W., Trim L., Plenderleith M.B., Beagley K.W. (2016). Induction of partial immunity in both males and females is sufficient to protect females against sexual transmission of Chlamydia. Mucosal Immunol..

[32] Hickey D.K., Aldwell F.E., Beagley K.W. (2009). Transcutaneous immunization with a novel lipid-based adjuvant protects against Chlamydia genital and respiratory infections. Vaccine.

[33] Wang S.P., Grayston J.T. (1963). Classification of Trachoma Virus Strains by Protection of Mice from Toxic Death. J. Immunol..

[34] Wang S.-P., Grayston J. (1984). Microimmunofluorescence serology of Chlamydia trachomatis. Medical Virology III.

[35] Wang S.P., Kuo C.C., Barnes R.C., Stephens R.S., Grayston J.T. (1985). Immunotyping of Chlamydia trachomatis with monoclonal antibodies. J. Infect. Dis..

[36] Tifrea D.F., Pal S., de la Maza L.M. (2020). A Recombinant Chlamydia trachomatis MOMP Vaccine Elicits Cross-serogroup Protection in Mice against Vaginal Shedding and Infertility. J. Infect. Dis..

[37] Tifrea D.F., Sun G., Pal S., Zardeneta G., Cocco M.J., Popot J.L., de la Maza L.M. (2011). Amphipols stabilize the Chlamydia major outer membrane protein and enhance its protective ability as a vaccine. Vaccine.

[38] Carmichael J.R., Pal S., Tifrea D., de la Maza L.M. (2011). Induction of protection against vaginal shedding and infertility by a recombinant Chlamydia vaccine. Vaccine.

[39] Murthy A.K., Li W., Guentzel M.N., Zhong G., Arulanandam B.P. (2011). Vaccination with the defined chlamydial secreted protein CPAF induces robust protection against female infertility following repeated genital chlamydial challenge. Vaccine.

[40] Tan C., Hsia R.C., Shou H., Haggerty C.L., Ness R.B., Gaydos C.A., Dean D., Scurlock A.M., Wilson D.P., Bavoil P.M. (2009). Chlamydia trachomatis-infected patients display variable antibody profiles against the nine-member polymorphic membrane protein family. Infect. Immun..

[41] Wang J., Zhang Y., Lu C., Lei L., Yu P., Zhong G. (2010). A genome-wide profiling of the humoral immune response to Chlamydia trachomatis infection reveals vaccine candidate antigens expressed in humans. J. Immunol..

[42] Cruz-Fisher M.I., Cheng C., Sun G., Pal S., Teng A., Molina D.M., Kayala M.A., Vigil A., Baldi P., Felgner P.L. (2011). Identification of immunodominant antigens by probing a whole Chlamydia trachomatis open reading frame proteome microarray using sera from immunized mice. Infect. Immun..

[43] Donati M., Sambri V., Comanducci M., Di Leo K., Storni E., Giacani L., Ratti G., Cevenini R. (2003). DNA immunization with pgp3 gene of Chlamydia trachomatis inhibits the spread of chlamydial infection from the lower to the upper genital tract in C3H/HeN mice. Vaccine.

[44] Luan X., Peng B., Li Z., Tang L., Chen C., Chen L., Wu H., Sun Z., Lu C. (2019). Vaccination with MIP or Pgp3 induces cross-serovar protection against chlamydial genital tract infection in mice. Immunobiology.

[45] Vasilevsky S., Stojanov M., Greub G., Baud D. (2016). Chlamydial polymorphic membrane proteins: Regulation, function and potential vaccine candidates. Virulence.

[46] Paes W., Brown N., Brzozowski A.M., Coler R., Reed S., Carter D., Bland M., Kaye P.M., Lacey C.J. (2016). Recombinant polymorphic membrane protein D in combination with a novel, second-generation lipid adjuvant protects against intra-vaginal Chlamydia trachomatis infection in mice. Vaccine.

[47] Muller T., Becker E., Stallmann S., Waldhuber A., Rommler-Dreher F., Albrecht S., Mohr F., Hegemann J.H., Miethke T. (2017). Vaccination with the polymorphic membrane protein A reduces Chlamydia muridarum induced genital tract pathology. Vaccine.

[48] Inic-Kanada A., Stojanovic M., Schlacher S., Stein E., Belij-Rammerstorfer S., Marinkovic E., Lukic I., Montanaro J., Schuerer N., Bintner N. (2015). Delivery of a Chlamydial Adhesin N-PmpC Subunit Vaccine to the Ocular Mucosa Using Particulate Carriers. PLoS ONE.

[49] Tan C., Hsia R.C., Shou H., Carrasco J.A., Rank R.G., Bavoil P.M. (2010). Variable expression of surface-exposed polymorphic membrane proteins in in vitro-grown Chlamydia trachomatis. Cell. Microbiol..

[50] Carrasco J.A., Tan C., Rank R.G., Hsia R.C., Bavoil P.M. (2011). Altered developmental expression of polymorphic membrane proteins in penicillin-stressed Chlamydia trachomatis. Cell. Microbiol..

[51] Nunes A., Gomes J.P., Mead S., Florindo C., Correia H., Borrego M.J., Dean D. (2007). Comparative expression profiling of the Chlamydia trachomatis pmp gene family for clinical and reference strains. PLoS ONE.

[52] Van Lent S., Creasy H.H., Myers G.S., Vanrompay D. (2016). The Number, Organization, and Size of Polymorphic Membrane Protein Coding Sequences as well as the Most Conserved Pmp Protein Differ within and across Chlamydia Species. J. Mol. Microbiol. Biotechnol..

[53] Crane D.D., Carlson J.H., Fischer E.R., Bavoil P., Hsia R.C., Tan C., Kuo C.C., Caldwell H.D. (2006). Chlamydia trachomatis polymorphic membrane protein D is a species-common pan-neutralizing antigen. Proc. Natl. Acad. Sci. USA.

[54] Kiselev A.O., Stamm W.E., Yates J.R., Lampe M.F. (2007). Expression, processing, and localization of PmpD of Chlamydia trachomatis Serovar L2 during the chlamydial developmental cycle. PLoS ONE.

[55] Swanson K.A., Taylor L.D., Frank S.D., Sturdevant G.L., Fischer E.R., Carlson J.H., Whitmire W.M., Caldwell H.D. (2009). Chlamydia trachomatis polymorphic membrane protein D is an oligomeric autotransporter with a higher-order structure. Infect. Immun..

[56] Favaroni A., Hegemann J.H. (2021). Chlamydia trachomatis Polymorphic Membrane Proteins (Pmps) Form Functional Homomeric and Heteromeric Oligomers. Front. Microbiol..

[57] Karunakaran K.P., Rey-Ladino J., Stoynov N., Berg K., Shen C., Jiang X., Gabel B.R., Yu H., Foster L.J., Brunham R.C. (2008). Immunoproteomic discovery of novel T cell antigens from the obligate intracellular pathogen Chlamydia. J. Immunol..

[58] Pal S., Favaroni A., Tifrea D.F., Hanisch P.T., Luczak S.E., Hegemann J.H., de la Maza L.M. (2017). Comparison of the nine polymorphic membrane proteins of Chlamydia trachomatis for their ability to induce protective immune responses in mice against a C. muridarum challenge. Vaccine.

[59] Peterson E.M., Markoff B.A., Schachter J., de la Maza L.M. (1990). The 7.5-kb plasmid present in Chlamydia trachomatis is not essential for the growth of this microorganism. Plasmid.

[60] Stothard D.R., Williams J.A., Van Der Pol B., Jones R.B. (1998). Identification of a Chlamydia trachomatis serovar E urogenital isolate which lacks the cryptic plasmid. Infect. Immun..

[61] Palmer L., Falkow S. (1986). A common plasmid of Chlamydia trachomatis. Plasmid.

[62] Galaleldeen A., Taylor A.B., Chen D., Schuermann J.P., Holloway S.P., Hou S., Gong S., Zhong G., Hart P.J. (2013). Structure of the Chlamydia trachomatis immunodominant antigen Pgp3. J. Biol. Chem..

[63] Chen D., Lei L., Lu C., Galaleldeen A., Hart P.J., Zhong G. (2010). Characterization of Pgp3, a Chlamydia trachomatis plasmid-encoded immunodominant antigen. J. Bacteriol..

[64] Li Z., Chen D., Zhong Y., Wang S., Zhong G. (2008). The chlamydial plasmid-encoded protein pgp3 is secreted into the cytosol of Chlamydia-infected cells. Infect. Immun..

[65] Li Z., Zhong Y., Lei L., Wu Y., Wang S., Zhong G. (2008). Antibodies from women urogenitally infected with C. trachomatis predominantly recognized the plasmid protein pgp3 in a conformation-dependent manner. BMC Microbiol..

[66] Li Z., Wang S., Wu Y., Zhong G., Chen D. (2008). Immunization with chlamydial plasmid protein pORF5 DNA vaccine induces protective immunity against genital chlamydial infection in mice. Sci. China C Life Sci..

[67] Tifrea D.F., Pal S., le Bon C., Cocco M.J., Zoonens M., de la Maza L.M. (2020). Improved protection against Chlamydia muridarum using the native major outer membrane protein trapped in Resiquimod-carrying amphipols and effects in protection with addition of a Th1 (CpG-1826) and a Th2 (Montanide ISA 720) adjuvant. Vaccine.

[68] Liu Y., Huang Y., Yang Z., Sun Y., Gong S., Hou S., Chen C., Li Z., Liu Q., Wu Y. (2014). Plasmid-encoded Pgp3 is a major virulence factor for Chlamydia muridarum to induce hydrosalpinx in mice. Infect. Immun..

[69] Pal S., Fielder T.J., Peterson E.M., de la Maza L.M. (1994). Protection against infertility in a BALB/c mouse salpingitis model by intranasal immunization with the mouse pneumonitis biovar of Chlamydia trachomatis. Infect. Immun..

[70] Peterson E.M., Zhong G.M., Carlson E., de la Maza L.M. (1988). Protective role of magnesium in the neutralization by antibodies of Chlamydia trachomatis infectivity. Infect. Immun..

[71] Atreya C.E., Ducker G.S., Feldman M.E., Bergsland E.K., Warren R.S., Shokat K.M. (2012). Combination of ATP-competitive mammalian target of rapamycin inhibitors with standard chemotherapy for colorectal cancer. Investig. New Drugs.

[72] Beal S.L. (2001). Ways to fit a PK model with some data below the quantification limit. J. Pharmacokinet. Pharmacodyn..

[73] Igietseme J.U., Eko F.O., Black C.M. (2011). *Chlamydia* vaccines: Recent developments and the role of adjuvants in future formulations. Expert Rev. Vaccines.

[74] Hafner L.M., Timms P. (2018). Development of a *Chlamydia trachomatis* vaccine for urogenital infections: Novel tools and new strategies point to bright future prospects. Expert Rev. Vaccines.

[75] de la Maza L.M., Pal S., Olsen A.W., Follmann F., Tan M., Hegemann J.H., Sutterlin C. (2020). Chlamydia Vaccines.

[76] Phillips S., Quigley B.L., Timms P. (2019). Seventy Years of Chlamydia Vaccine Research—Limitations of the Past and Directions for the Future. Front. Microbiol..

[77] Kaufhold R.M., Boddicker M.A., Field J.A., Lucas B.J., Antonello J.M., Espeseth A.S., Skinner J.M., Heinrichs J.H., Smith J.G. (2019). Evaluating potential vaccine antigens in both the Chlamydia trachomatis and Chlamydia muridarum intravaginal mouse challenge models. World J. Vaccines.

[78] Saka H.A., Thompson J.W., Chen Y.S., Kumar Y., Dubois L.G., Moseley M.A., Valdivia R.H. (2011). Quantitative proteomics reveals metabolic and pathogenic properties of Chlamydia trachomatis developmental forms. Mol. Microbiol..

[79] Sharma J., Zhong Y., Dong F., Piper J.M., Wang G., Zhong G. (2006). Profiling of human antibody responses to Chlamydia trachomatis urogenital tract infection using microplates arrayed with 156 chlamydial fusion proteins. Infect. Immun..

[80] Comanducci M., Manetti R., Bini L., Santucci A., Pallini V., Cevenini R., Sueur J.M., Orfila J., Ratti G. (1994). Humoral immune response to plasmid protein pgp3 in patients with Chlamydia trachomatis infection. Infect. Immun..

[81] Qiu S., Zhang J., Tian Y., Yang Y., Huang H., Yang D., Lu M., Xu Y. (2008). Reduced antigenicity of naturally occurring hepatitis B surface antigen variants with substitutions at the amino acid residue 126. Intervirology.

[82] Yu H., Jiang X., Shen C., Karunakaran K.P., Jiang J., Rosin N.L., Brunham R.C. (2010). Chlamydia muridarum T-cell antigens formulated with the adjuvant DDA/TDB induce immunity against infection that correlates with a high frequency of gamma interferon (IFN-gamma)/tumor necrosis factor alpha and IFN-gamma/interleukin-17 double-positive CD4+ T cells. Infect. Immun..

[83] Finco O., Frigimelica E., Buricchi F., Petracca R., Galli G., Faenzi E., Meoni E., Bonci A., Agnusdei M., Nardelli F. (2011). Approach to discover T- and B-cell antigens of intracellular pathogens applied to the design of Chlamydia trachomatis vaccines. Proc. Natl. Acad. Sci. USA.

[84] Coler R.N., Bhatia A., Maisonneuve J.F., Probst P., Barth B., Ovendale P., Fang H., Alderson M., Lobet Y., Cohen J. (2009). Identification and characterization of novel recombinant vaccine antigens for immunization against genital *Chlamydia trachomatis*. FEMS Immunol. Med. Microbiol..

[85] Qiu Q., Wang R.Y., Jiao X., Jin B., Sugauchi F., Grandinetti T., Alter H.J., Shih J.W. (2008). Induction of multispecific Th-1 type immune response against HCV in mice by protein immunization using CpG and Montanide ISA 720 as adjuvants. Vaccine.

[86] Cheng C., Jain P., Pal S., Tifrea D., Sun G., Teng A.A., Liang X., Felgner P.L., de la Maza L.M. (2014). Assessment of the role in protection and pathogenesis of the Chlamydia muridarum V-type ATP synthase subunit A (AtpA) (TC0582). Microbes Infect..

[87] Li W., Guentzel M.N., Seshu J., Zhong G., Murthy A.K., Arulanandam B.P. (2007). Induction of cross-serovar protection against genital chlamydial infection by a targeted multisubunit vaccination approach. Clin. Vaccine Immunol..

[88] Tifrea D.F., Barta M.L., Pal S., Hefty P.S., de la Maza L.M. (2016). Computational modeling of TC0583 as a putative component of the Chlamydia muridarum V-type ATP synthase complex and assessment of its protective capabilities as a vaccine antigen. Microbes Infect..

[89] Chen C., Zhong G., Ren L., Lu C., Li Z., Wu Y. (2015). Identification of Plasmid-Free Chlamydia muridarum Organisms Using a Pgp3 Detection-Based Immunofluorescence Assay. J. Microbiol. Biotechnol..

