# Supplementary material for: The Polymorphic Membrane Protein G Has a Neutral Effect and the Plasmid Glycoprotein 3 an Antagonistic Effect on the Ability of the Major Outer Membrane Protein to Elicit Protective Immune Responses against a Chlamydia muridarum Respiratory Challenge
Source: Vaccines (Basel). 2023 Feb 21;11(3):504. doi: 10.3390/vaccines11030504 (PMC10051784; doi:10.3390/vaccines11030504)
Supplement: Supplementary file 1 [file vaccines-11-00504-s001.zip › vaccines-2133277-supplementary.pdf]

vaccines

MDPI

Supplementary Materials

Table S1. Vaccine induced antibody responses in sera the day before the *C. muridarum* intranasal challenge.

Immunization groups	Anti EB serum GMT (range)		IgG2a/IgG1 ratio	Neutralizing Ab GMT (range)
	IgG1	IgG2a		
Pgp3	1131 [a,b,c,d] (200–12,800)	9870 [a,b,c,d] (6400–12,800)	8.7	<50 (<50 – <50)
PmpG	436 [a,b,c,d] (200–800)	6400 [a,b,c,d] (3200–12,800)	14.7	<50 (<50 – <50)
MOMP + Pgp3	23,475 [a,b] (12,800–51,200)	223,336 [a] (102,400–409,600)	9.5	79 (<50 – 800)
MOMP + PmpG	39,481 [a] (12,800–102,400)	265,593 [a] (102,400–819,200)	6.7	63 (<50 – 400)
MOMP	64,508 [a] (12,800–102,400)	409,600 [a] (102,400–1,638,400)	6.4	159 (<50 – 800)
PBS	<100	<100	-	<50

GMT: Geometric mean titer. [a] $P < 0.05$ by the Mann-Whitney's *U*-test compared with the PBS group. [b] $P < 0.05$ by the Mann-Whitney's *U*-test compared with the MOMP group. [c] $P < 0.05$ by the Mann-Whitney's *U*-test compared with the MOMP + PmpG group. [d] $P < 0.05$ by the Mann-Whitney's *U*-test compared with the MOMP + Pgp3 group.

Table S2. Vaccine induced antibody responses in sera the day before the *C. muridarum* intranasal challenge.

Immunization groups	IgG GMT (range) in sera against Pgp3, PmpG and MOMP (x10^3)		
	MOMP	Pgp3	PmpG
Pgp3	n/m	2826.5 [a] (1,280.0–5,120.0)	n/m
PmpG	n/m	n/m	905.1 [a] (640.0–1,280.0)
MOMP + Pgp3	69.6 [a,b] (40.0–80.0)	2560.0 [a] (2,560.0–2,560.0)	n/m
MOMP + PmpG	89.8 [a] (80.0–160.0)	n/m	543.5 [a,c] (320.0–640.0)
MOMP	139.9 [a] (40.0–409.6)	n/m	n/m
PBS	<100	<100	<100

GMT: Geometric mean titer. n/m: Not measured. [a] $P < 0.05$ by the Mann-Whitney's *U*-test compared with the PBS group. [b] $P < 0.05$ by the Mann-Whitney's *U*-test compared with the MOMP group. [c] $P < 0.05$ by the Mann-Whitney's *U*-test compared with the PmpG group.

Table S3. *C. muridarum*-specific IgG and IgA titers in vaginal washes the day before the *C. muridarum* i.n. challenge.

Immunization groups	Anti-EB titer	
	IgG	IgA
Pgp3	<10	<10
PmpG	<10	10
MOMP + Pgp3	160	10
MOMP + PmpG	640	20
MOMP	640	20
PBS	<10	<10

Table S4. In vitro cytokine production by T cells from immunized mice the day before challenge.

Immunization group	EB stimulated		ConA stimulated	
	IFN-γ (pg/ml) (mean ± 1 SE)	IL-4 (pg/ml) (mean ± 1 SE)	IFN-γ (pg/ml) (mean ± 1 SE)	IL-4 (pg/ml) (mean ± 1 SE)
Pgp3	<15 ± <15 [a,b,c]	<4 ± <4 [a,b]	10,458.13 ± 1990.84	86.82 ± 9.86
PmpG	<15 ± <15 [a,b,c]	<4 ± <4 [a,b]	5891.12 ± 1815.75	39.43 ± 6.13
MOMP + Pgp3	60.70 ± 41.69 [a,b,d]	<4 ± <4 [a,b]	11,845.08 ± 1218.38	136.35 ± 50.19
MOMP + PmpG	811.38 ± 411.17 [d]	4.43 ± 1.61 [d]	13,744.70 ± 2255.65	143.28 ± 37.08

MOMP	796.05 ± 530.32[d]	9.59 ± 6.73[d]	9032.99 ± 1550.93	112.48 ± 11.42
PBS	<15 ± <15	<4 ± <4	14,104.43 ± 2,481.78	87.58 ± 14.58

[a] $P < 0.05$ by the Student's *t*-test compared with the MOMP group. [b] $P < 0.05$ by the Student's *t*-test compared with the MOMP + PmpG group. [c] $P < 0.05$ by the Student's *t*-test compared with the MOMP + Pgp3 group. [d] $P<0.05$ by the Student's *t*-test compared with the PBS group.
